# Right Atrial Ablation for Premature Ventricular Contractions Originating From the Posterior‐Superior Process of the Left Ventricle: A Novel Therapeutic Approach

**DOI:** 10.1002/joa3.70186

**Published:** 2025-09-09

**Authors:** Yuki Kato, Yuichiro Sagawa, Kazuya Murata, Tetsuo Sasano, Yasuteru Yamauchi

**Affiliations:** ^1^ Department of Cardiology Japan Red Cross Yokohama City Bay Hospital Kanagawa Japan; ^2^ Department of Cardiovascular Medicine, Institute of Science Tokyo Japan

**Keywords:** atrioventricular septum, catheter ablation, premature ventricular contractions, right atrial ablation, ventricular tachycardia arrhythmia

## Abstract

We experienced a case of premature ventricular contractions originating from the posterior‐superior process of the left ventricle that was successfully ablated from the right atrium where the A/V ratio was 5:1.

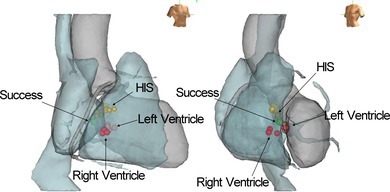

## 1 Introduction

Catheter ablation is difficult for premature ventricular contractions (PVCs) originating from the basal septum of the ventricles [1]. One reason for this is the proximity of the ablation site to the His bundle recording site. Herein, we present a case of PVCs successfully eliminated by radiofrequency ablation from the right atrium (RA) after failure of ablation from the left ventricle (LV).

## 2 Case Report

**FIGURE 1:**
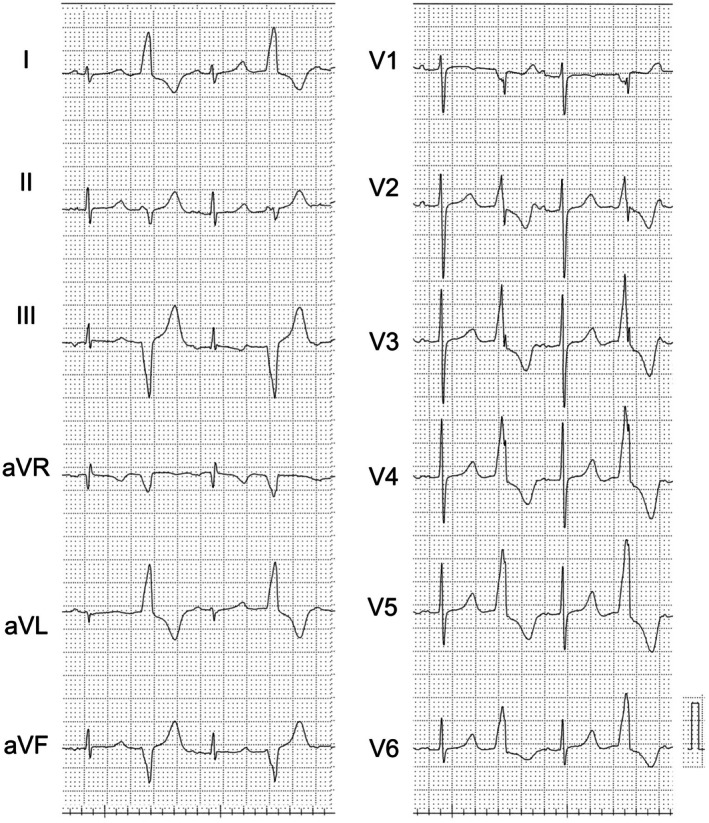
Twelve‐lead electrocardiogram showing the morphology of the premature ventricular contraction (PVCs). The PVC exhibited a left bundle branch block pattern. Lead I showed an R wave, lead II showed an rS pattern, lead III and V_1_ showed a QS pattern, and a precordial transitional zone was observed in leads V1‐V2. These findings suggested that the PVC originated from the posterior‐superior process of the left ventricle.

A 60‐year‐old woman with no notable medical history presented with chest discomfort during periods of stress. A Holter electrocardiogram monitor revealed a high burden of PVCs at 29380 beats/day (26%). Despite administering beta‐blocker therapy, repeated Holter monitoring showed persistent PVCs at 31389 beats/day (28%). Twelve‐lead eclectrocardiogram of the PVCs showed an R wave in lead I, an rS pattern in lead II, a QS pattern in leads III and V1, and the precordial transition zone was observed in leads V1‐2 (Figure 1). The PVC was suspected to have originated from the atrioventricular valve annulus in the midventricular septum. Echocardiography revealed a left ventricular ejection fraction of 60%.

**FIGURE 2:**
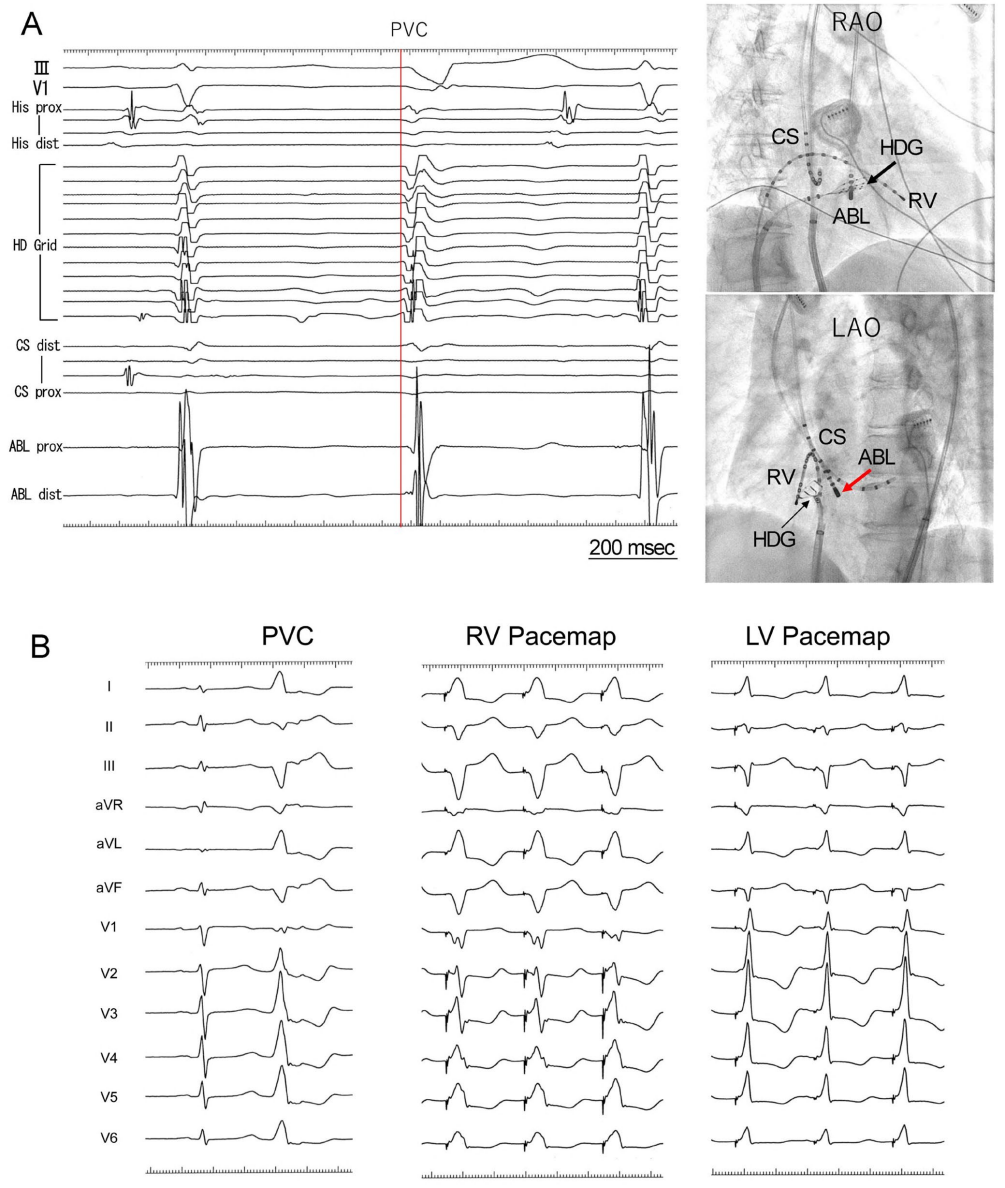
Illustrations showing the evaluation of the origin of the premature ventricular contraction (PVCs). (A) Intracardiac electrogram placement and findings. The HD Grid (HDG) catheter was positioned on the right ventricular (RV) septum and an ablation catheter (ABL) on the left ventricular (LV) septum. The earliest activation site was identified on the RV side, whereas the LV side exhibited a slightly delayed activation. The black and red arrows indicate the HDG and ABL catheters, respectively. (B) Pace mapping on both sides of the LV. The limb leads demonstrated a morphology consistent with clinical PVC on both the RV and LV sides. In the precordial leads, the transition zone was located at lead V3 on the RV side and lead V1 on the LV side. Pace mapping was performed in the RV using an output of 9.9 V / 2.0 ms, whereas capture in the LV was achieved at a lower output of 5.0 V / 2.0 ms.

**FIGURE 3:**
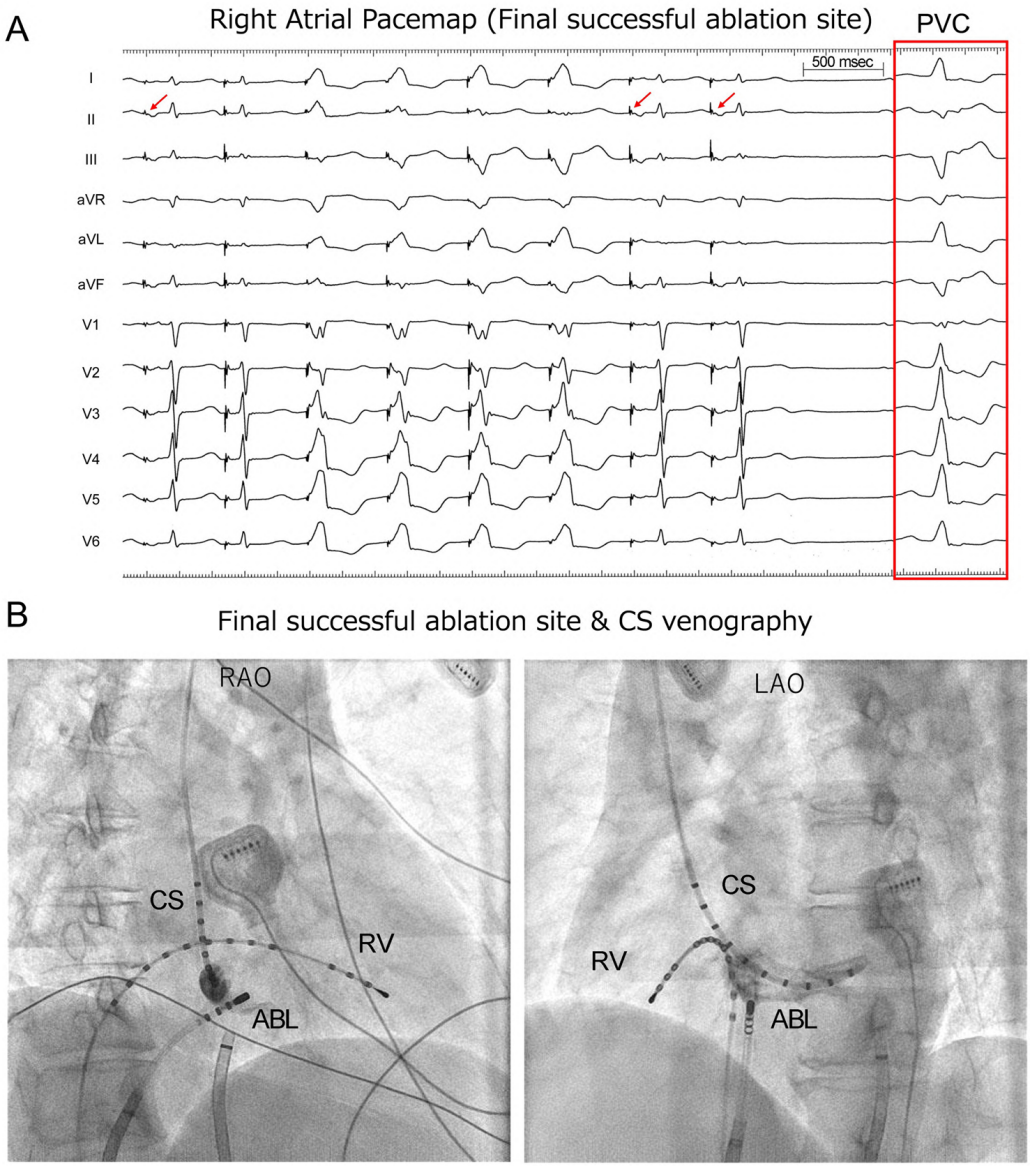
Pace mapping findings at the final successful right atrial ablation site, along with a cineangiogram obtained during coronary sinus venography (RA). (A) Pacing near the coronary sinus (CS) ostium in the RA produced a QRS morphology that closely resembled that of the clinical PVC, although the precordial transition zone was slightly different. Atrial capture was also observed at this site, as indicated by the red arrows. Pace mapping was performed in the RA using an output of 9.9 V/2.0 msec. (B) A representative fluoroscopic image taken at the final successful ablation site with contrast injection into the CS ostium. ABL, ablation catheter; CS, coronary sinus; HDG, HD grid; LAO, left anterior oblique; RAO, right anterior oblique; RV, right ventricle.

**FIGURE 4:**
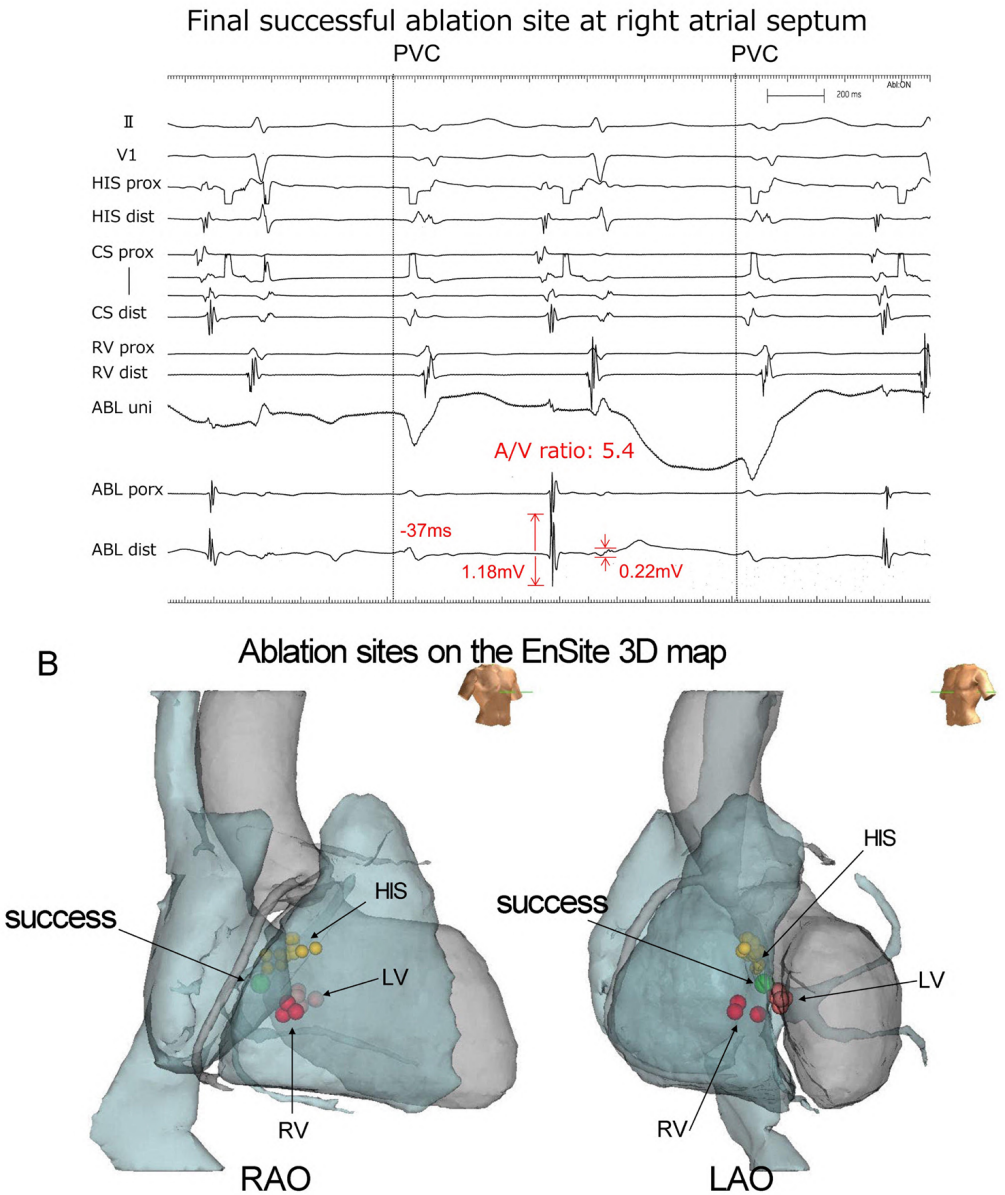
Intracardiac electrograms at successful ablation site and corresponding ablation site on three‐dimensional electroanatomical mapping. (A) Intracardiac electrograms recorded at the successful ablation site in the right atrium. During the PVC, a sharp potential preceding a QRS onset of 37 ms was observed on the distal pair of the ablation catheter. The unipolar signal at this site exhibited a QS pattern. During sinus rhythm, the atrial‐to‐ventricular amplitude ratio was 5.4, with a distinctly larger A wave than the V wave. Due to the overlap and interference between the electrodes during the procedure, it was technically difficult to remove the “HIS prox” recording and the second line with the “CS prox” recording of large artifacts. (B) Representative image of the ablation site on the three‐dimensional electroanatomical mapping. The successful ablation point was located on the right atrial side, opposite the left ventricular site, where transient suppression of PVCs was observed. This site was situated directly beneath the His bundle. HIS, His bundle; LAO, left anterior oblique; LV, left ventricle; RAO, right anterior oblique; RV, right ventricle.

Electrophysiological analysis and radiofrequency catheter ablation targeting of PVCs were performed using a three‐dimensional electroanatomical mapping system (EnSite system). The site of origin of the PVC was determined based on detailed activation and pace mapping. Activation mapping revealed that the basal region near the right ventricular (RV) septum was the earliest site of activation. When the HD Grid mapping catheter was positioned in the RV septum and the ablation (ABL) catheter was placed in the LV septum to confirm the intracardiac electrograms, the earliest activation site was identified on the RV side, with a slight delay observed in the LV ventricle (Figure 2A). During pace mapping, the limb leads on the RV side closely matched clinical PVCs. Within the chest leads, the transition zone during RV pace mapping was observed at lead V3, whereas during LV pace mapping, it was located at lead V1 (Figure 2B). For safety reasons, we first ablated the basal septum of the left ventricle. The PVCs transiently disappeared, and it took approximately 50 s for the PVCs to be eliminated, but they reappeared a few minutes after the radiofrequency energy was turned off. Next, radiofrequency energy was delivered to the basal septum of the right ventricle, the earliest activation site within the right ventricle and the anatomical counterpart of the left ventricular basal septum where transient ablation had previously been effective; however, this ablation attempt was ineffective. When pace mapping was performed in the RA near the coronary sinus ostium, the paced QRS morphology closely resembled that of clinical PVCs, although the transition zone was slightly different. Simultaneously, atrial capture was confirmed (Figure 3A,B). During the PVC, the intracardiac electrogram recorded a potential at the distal end of the ABL catheter that preceded the QRS complex by 37 ms. In addition, a QS pattern was observed in the unipolar recording. At the site of successful ablation during sinus rhythm, the atrial‐to‐ventricular (A/V) ratio was 5.4, and a prominent A wave was observed, which was greater than the V wave (Figure 4A). After ablation at the same site, the PVCs disappeared within a few seconds. Postoperative Holter monitoring also showed a significant reduction in PVCs to nine beats/day. The transient disappearance of PVCs occurred at the ablation site in the LV, which was precisely opposite to the successful ablation site located just beneath the His bundle in the RA (Figure 4B). The patient has remained free of PVC recurrence for more than 1 year.

## 3 Discussion

We encountered a case in which an RA approach was successful for PVCs originating from the interventricular septum despite ineffective endocardial ablation from the LV and RV. Ventricular arrhythmias originating from the left posterior‐superior process of the LV (PSP‐LV) can be ablated from the opposite RA. The disappearance of PVCs after the initiation of RF energy varied depending on the ablation site. When the ablation catheter was positioned in the left ventricle, it took approximately 50 s for the PVCs to be eliminated. In contrast, RF energy delivery from the right atrium resulted in PVC disappearance within a few seconds. This rapid response suggests that the site of origin was anatomically closer to the right atrial aspect of the PSP‐LV. Santangeli et al. reported five cases of successful ablation of PVCs originating from the PSP‐LV; however, in all five cases, the A wave was smaller than the V wave [2]. In this case, electrical stimulation successfully achieved ventricular capture at a site where the A/V ratio was 5:1, as well as atrial capture (Figure 3A). Arai et al. reported a case in which a PVC was successfully treated with ablation from the RA side of the atrioventricular septum [3]. This case is similar to ours in that it involved a superior axis left bundle branch block morphology with a transition zone in leads V1‐V2. There are also reports describing the electrocardiogram characteristics of PVCs originating from the same site, as well as cases in which they disappeared from the inferoseptal recess following ablation [4]. On pace mapping, the LV site demonstrated a higher score. The pre‐potential preceded the QRS complex by 45 ms. Although the PVCs were reduced with ablation at the septal LV site, they did not completely disappear. Furthermore, at the successful ablation site in the RA, the A/V ratio during sinus rhythm was approximately 0.3. This A/V ratio differs from that observed in our study. Martinov et al. reported cases similar to ours. They reported a case in which pacing from the RA was successful at an A/V ratio of 5, consistent with our case [5]. However, our case differs from their report in two points: in our study, the PVCs temporarily disappeared during LV ablation, and atrial capture was observed at the successful ablation site. The key findings were the presence of early activation and the capture of both atrial and ventricular electrograms during pacing. Ventricular capture was essential to confirm proximity to the PVC origin, and ablation would not have been attempted if only atrial capture was observed.

## 4 Conclusion

We report a case of PVCs originating from the interventricular septum that was successfully ablated using an RA approach. Although previous reports described ablation targeting the PSP‐LV, our patient demonstrated distinct electrocardiographic and intracardiac features. To the best of our knowledge, this is the first report of successful ablation with complete atrial capture at an A/V ratio of ≥ 1. These findings highlight the potential of an RA approach as a viable alternative for the PSP‐LV of PVCs when conventional strategies fail.

## Consent

Written informed consent for the submission and publication of this case report, including all accompanying images and text, was obtained from the patient in accordance with the guidelines of the Committee on Publication Ethics (COPE).

## Conflicts of Interest

The authors declare no Conflicts of Interest.

## Data Availability

Data supporting the findings of this study are available from the corresponding author upon reasonable request.

## References

[1] P. Futyma , R. Głuszczyk , M. Futyma , and P. Kułakowski , “Right Atrial Position of a Return Electrode for Bipolar Ablation of the Left Posterosuperior Process Ventricular Tachycardia,” Pacing and Clinical Electrophysiology 42 (2019): 474–477, 10.1111/pace.13554. 30461031

[2] P. Santangeli , M. D. Hutchinson , G. E. Supple , D. J. Callans , F. E. Marchlinski , and F. C. Garcia , “Right Atrial Approach for Ablation of Ventricular Arrhythmias Arising From the Left Posterior‐Superior Process of the Left Ventricle,” Circulation. Arrhythmia and Electrophysiology 9 (2016): e004048, 10.1161/CIRCEP.116.004048. 27412430

[3] M. Arai , S. Fukamizu , I. Kawamura , et al., “Successful Catheter Ablation of Ventricular Premature Complexes From the Right Atrial Side of the Atrioventricular Septum With Good Contact Force,” J Arrhythm 34 (2018): 201–203, 10.1002/joa3.12038. 29657597 PMC5891414

[4] A. Li , Z. Zuberi , J. S. Bradfield , et al., “Endocardial Ablation of Ventricular Ectopic Beats Arising From the Basal Inferoseptal Process of the Left Ventricle,” Heart Rhythm 15 (2018): 1356–1362, 10.1016/j.hrthm.2018.04.029. 29709577

[5] E. Martinov , D. Marchov , M. Marinov , D. Boychev , V. Gelev , and V. Traykov , “Endocardial, Epicardial, and Right Atrial Approach for Catheter Ablation of Premature Ventricular Contractions From the Inferoseptal Process of the Left Ventricle,” Journal of Arrhythmia 39 (2023): 613–620, 10.1002/joa3.12870. 37560291 PMC10407169

